# AAV-Mediated, Optogenetic Ablation of Müller Glia Leads to Structural and Functional Changes in the Mouse Retina

**DOI:** 10.1371/journal.pone.0076075

**Published:** 2013-09-27

**Authors:** Leah C. Byrne, Fakhra Khalid, Trevor Lee, Emilia A. Zin, Kenneth P. Greenberg, Meike Visel, David V. Schaffer, John G. Flannery

**Affiliations:** 1 Department of Molecular and Cellular Biology and The Helen Wills Neuroscience Institute, University of California, Berkeley, California, United States of America; 2 Spiral Devices, LLC, Berkeley, California, United States of America; 3 Department of Chemical Engineering, Department of Bioengineering, and The Helen Wills Neuroscience Institute, University of California, Berkeley, California, United States of America; Columbia University, United States of America

## Abstract

Müller glia, the primary glial cell in the retina, provide structural and metabolic support for neurons and are essential for retinal integrity. Müller cells are closely involved in many retinal degenerative diseases, including macular telangiectasia type 2, in which impairment of central vision may be linked to a primary defect in Müller glia. Here, we used an engineered, Müller-specific variant of AAV, called ShH10, to deliver a photo-inducibly toxic protein, KillerRed, to Müller cells in the mouse retina. We characterized the results of specific ablation of these cells on visual function and retinal structure. ShH10-KillerRed expression was obtained following intravitreal injection and eyes were then irradiated with green light to induce toxicity. Induction of KillerRed led to loss of Müller cells and a concomitant decrease of Müller cell markers glutamine synthetase and cellular retinaldehyde-binding protein, reduction of rhodopsin and cone opsin, and upregulation of glial fibrillary acidic protein. Loss of Müller cells also resulted in retinal disorganization, including thinning of the outer nuclear layer and the photoreceptor inner and outer segments. High resolution imaging of thin sections revealed displacement of photoreceptors from the ONL, formation of rosette-like structures and the presence of phagocytic cells. Furthermore, Müller cell ablation resulted in increased area and volume of retinal blood vessels, as well as the formation of tortuous blood vessels and vascular leakage. Electrophysiologic measures demonstrated reduced retinal function, evident in decreased photopic and scotopic electroretinogram amplitudes. These results show that loss of Müller cells can cause progressive retinal degenerative disease, and suggest that AAV delivery of an inducibly toxic protein in Müller cells may be useful to create large animal models of retinal dystrophies.

## Introduction

Müller glia are the principal glial cell in the retina and provide structural and metabolic support to all neuronal cell types. Müller cells have soma that lie in the inner nuclear layer (INL) and radial processes that span the retina from the inner limiting membrane (ILM) to the outer limiting membrane (OLM). Müller cell endfeet in the ILM contact the vitreous, while adherens junctions between Müller cells and photoreceptors form the OLM. Müller glia are also closely involved with the development and maintenance of the retinal vasculature. Müller cell endfeet contact large retinal blood vessels at the inner surface of the retina and thereby participate in the formation of the blood-retinal barrier. Müller cell processes wrap around retinal capillaries along with endothelial cells, and secretion of factors such as PEDF and GDNF from Müller cells has been shown to increase blood-retina barrier integrity [1,2].

Müller glia perform a multitude of important regulatory and supportive roles in the retina, including secretion of trophic factors, as well as removal of metabolic waste, neurotransmitter recycling and regulation of K^+^ and water homeostasis [3,4]. Müller cells take up glutamate through the glutamate/aspartate transporter; neurotransmitter recycling then occurs through glutamine synthetase (GS) -catalyzed conversion of the neurotransmitter glutamate to glutamine.

Müller cell reactivity, or gliosis, occurs in nearly all retinal pathologies. Under conditions of damage or disease, Müller cells upregulate the intermediate filaments glial fibrillary acidic protein (GFAP), vimentin and nestin. Müller cells can proliferate, form a glial scar, and release inflammatory cytokines, thereby inducing further loss of retinal cells. Additionally, the release of VEGF from Müller cells in response to injury results in neovascularization and leakage of blood vessels.

Dysregulation or loss of Müller cell function may be a primary cause of some retinal degenerations, including macular telangiectasia type 2 (MacTel-2) [5], which is characterized by intraretinal vascular changes, loss of vision, and reduced macular pigment. MacTel-2 is relatively rare, affecting approximately 0.1% of the population, though the actual incidence may be higher since MacTel-2 is poorly understood and conclusive diagnosis is thus difficult [6]. In a post-mortem study of a MacTel-2 donor eye, immunocytochemistry showed a reduction of Müller cell markers in the macula, including GS and vimentin, and this region also exhibited a loss of central macular pigment [5]. These changes were confirmed by a proteomics study of the fellow eye that showed a reduction in Müller cell-associated proteins [7].

Müller cell ablation has been used to examine the potential role of Müller cell dysfunction as a cause of diseases such as MacTel-2. Müller cell death in transgenic mice overexpressing the human Bcl-2 protein caused retinal dysplasia, photoreceptor apoptosis and retinal degeneration [8]. In another study, subretinal injection of the glial toxin DL-α-aminoadipic acid disrupted retinal glial cells in the rat retina, induced vascular telangiectasis and increased vascular permeability up to 4 months post-injection [9], though the toxin did not eliminate Müller cells after subretinal injection in the primate retina [10]. Finally, a transgenic mouse model with conditional expression of an attenuated diphtheria toxin under the control of a Müller cell-specific promoter eliminated Müller glia and led to severe and rapid photoreceptor apoptosis, vascular changes, breakdown of the blood-retina barrier and subretinal neovascularization [11].

While previous studies have established the importance of Müller cells in retinal function, they cannot be readily translated from rodents to a large animal model more representative of human retina. The human retina contains a macula, which harbors a high concentration of cones as well as a specialized population of Müller glia, while rodents lack this anatomical feature. MacTel-2 specifically affects the macula in humans, and therefore a rodent model may be insufficient to understand the underlying disease mechanisms. It is important to create a method of Müller cell elimination that can also be used in large animals with eye structure similar to humans. Here, we test a viral vector-mediated approach to deliver an inducibly phototoxic protein, KillerRed, to Müller glia in rodents – which may be used in future experiments in primates – and characterize the changes that occur in the retina as a result of specific elimination of these cells. These results demonstrate the importance of Müller cells to the integrity of the retina and indicate that the changes in retinal structure and function observed in retinal degenerative diseases such as MacTel-2 may be linked to Müller cell dysfunction.

## Materials and Methods

### Ethics Statement

All procedures were performed in accordance with the ARVO statement for the Use of Animals in Ophthalmic and Vision Research, and were approved by the University of California Animal Care and Use Committee AUP# R200-0913BC. All surgery was performed under anesthesia, and all efforts were made to minimize suffering.

### Viral vectors

AAV vectors were produced using a plasmid triple transfection method [12]. Recombinant AAV was purified using iodixanol gradient ultracentrifugation, and DNase resistant genomes were titered by quantitative PCR relative to standards. Each vector contained a self-complimentary genome encoding GFP or KillerRed under the control of a ubiquitous CAG promoter. KillerRed cDNA was amplified from the pKillerRed-mem vector (Evrogen, Moscow, Russia). The vectors were further modified with a single Y445F tyrosine to phenylalanine mutation for enhanced intracellular and nuclear trafficking [13], which was introduced into the ShH10 capsid plasmid using a site directed mutagenesis kit (QuikChange Lightning, Agilent Technologies).

### Animals

C57BL/6 mice from Jackson Laboratories were used for all experiments. Mice were housed in a 12 hour light/dark cycle, except for animals used for antibody labeling of KillerRed, which were housed in constant darkness for the duration of the experiment.

### Intravitreal injections

Mice were anesthetized with ketamine (58 mg/kg) and xylazine (6.5 mg/kg) by intraperitoneal injection. An ultrafine 30 1/2-gauge disposable needle was then passed through the sclera, at the equator and next to the limbus, into the vitreous cavity. Two µL containing 2 × 10^13^ vg/ml of AAV were injected with direct observation of the needle in the center of the vitreous cavity above the optic nerve head.

### Light exposure

One month after intravitreal injections with ShH10-KillerRed and ShH10-GFP, we placed 5 mice in a reflective metal cylindrical container after dilating eyes with phenylephrine (2.5%) and tropicamide (1%). We placed a green LED light source (540-580 nm) covering the container, adjusting it so that the light reaching the mice had an intensity of 1000 lux. Mice were irradiated for 1 hour per irradiation session. Irradiations were repeated for a total exposure time of at least 3 hours, with all irradiations performed on separate days. For time course experiments mice were irradiated for 2 hours in a single session.

### Immunohistochemistry

One to five months after irradiation, animals were humanely euthanized and eyecups were fixed in 4% paraformaldehyde overnight before being embedded in 5% agarose. One hundred micron transverse sections were cut on a vibratome in a PBS bath. Sections were briefly post-fixed, rinsed in PBS and blocked in 1% bovine serum albumin, 0.5% Triton X-100, and 2% normal donkey serum for 2–3 hours before antibody labeling overnight at 4°C. Antibodies were: PNA (Molecular Probes, 1:200), anti-M-opsin (Chemicon International, 1:500), anti-glutamine synthetase (Sigma, 1:1000), anti-GFAP (DAKO A/S, 1:1000), anti-laminin (Sigma, 1:50), anti-KillerRed (Evrogen, 1:1000). After rinsing in PBS, section were incubated with secondary antibodies conjugated to Alexa-488 or Alexa-594 for two hours at RT. Sections were rinsed and mounted with medium containing DAPI. Sections were then imaged on a confocal microscope (Carl Zeiss LSM710; Carl Zeiss Microimaging, Peabody, MA, USA).

### Thin sectioning

Animals were perfused with paraformaldehyde/glutaraldehyde, and enucleated. Eyes were then fixed in osmium tetroxide, dehydrated in ethanol solutions of increasing concentration, and embedded in resin. Eye cups were sectioned at 1 micron thickness through the superior/inferior axis. Slides were stained in toluene blue on a heated surface and rinsed in distilled water before mounting with Permount (Fisher Scientific).

### Spectral domain-optical coherence tomography (SD-OCT)

Images of retinal cross-sections were averaged from 8 contiguous slices. Histological imaging was performed using an 840 nm SDOIS OCT system (Bioptigen, Durham, North Carolina). Retinal thickness, ONL thickness, and photoreceptor inner and outer segment length measurements were gathered using segmentation tools in InVivoVue software. In regions of disorganization, thicknesses were measured by increasing contrast in the images in order to visualize a breakpoint between layers. Images were quantified separately by two investigators to confirm the analysis.

### qRT-PCR

Animals were humanely euthanized, and retinas was collected from n=5 mice, which were injected in one eye with ShH10-KillerRed or ShH10-GFP in the contralateral eye. RNA was extracted and subjected to DNase digestion, and the resulting RNA was used to create cDNA. qRT-PCR was performed on samples in triplicate using validated primers [14,15] and using the housekeeping gene glyceraldehyde-3-phosphate dehydrogenase as an internal control. The relative standard curve method was used to calculate fold difference of expression normalized to control eyes. Uninjected wild-type mouse retinas (n=5) served as a negative control. Primers were: CRALBP F: tgctggaaaatgaggaaacc; CRALBP R: CAAGAAGGGCTTGACCACAT; GFAP F: AAGCTCCAAGATGAAACCAACCTGA; GFAP R: CTTGGCCACATCCATCTCCA; GNAT2 F: gcatcagtgctgaggacaaa; GNAT2 R: ctaggcactcttcgggtgag; GLUI F: GGATAGCCCGTTTTATCTTGC; GLU1 R: GTGGTACTGGTGCCTCTTGC; Rhodopsin F: caagaatccactgggagatga; Rhodopsin R: gtgtgtggggacaggagact; VEGF F2: CTGTGCAGGCTGCTGTAACG VEGF R2: GTTCCCGAAACCCTGAGGAG; mGAPDHf1: TGCCCCCATGTTTGTGAT; mGAPDHr1: TGTGGTCATGAGCCCTTCC.

### Electroretinogram (ERG) recordings

Scotopic ERGs were recorded (Espion E2 ERG system; Diagnosys LLC, Littleton, MA) in response to six light flash intensities ranging from −3 to 1 log cd x s/m^2^ on a dark background. Each stimulus was presented in series of three. For photopic ERGs the animal was first exposed to a rod-saturating background for 10 minutes. Stimuli ranging from -0.9 to 1.4 log cd x s/m^2^ were presented 20 times on a lighted background. Data were analyzed with MatLab (v7.7; Mathworks, Natick, MA), and ERG amplitudes were compared using a paired two-tailed Student’s t-test.

### DiI perfusion and quantification

Mice were perfused with DiI as described previously [16]. Labeled retinas were then flatmounted or embedded in agarose and sectioned at 100 microns, and imaged on a confocal microscope (Carl Zeiss LSM710; Carl Zeiss Microimaging, Peabody, MA, USA). Quantification of blood vessel area and volume was performed on 10X confocal stacks centered on the optic nerve head using volume and area tools in Imaris software (Bitplane). Identical parameters were used to quantify all samples.

### Fluorescein imaging

Leakage from retinal blood vessels in mice was visualized by intraperitoneal injection of sodium fluorescein (Sigma Aldrich) in 0.1 ml sterile saline. Images were collected after injection using a fundus camera (Micron II; Phoenix Research Labs Inc., Pleasanton, CA, USA). Pupils were dilated for fundus imaging with phenylephrine (2.5%) and atropine sulfate (1%).

### Glutamine synthetase labeling and quantification

Agarose-embedded retinas were sectioned and labeled with anti-GS antibodies, followed by labeling with a secondary Alexa-594 antibody. 10X images were collected using equal acquisition parameters for all samples from across the entire retinal section, and then a montage of these images was created by overlapping images. Imaris software (Bitplane, Zurich, Switzerland) was used to quantify the total area of the retinal section and the area containing GS labeling, above the threshold of background. The area containing labeling was normalized to the total area of the retina.

### Analysis of cell death

Eyecups were immersion fixed, placed in sucrose overnight, then frozen in OCT embedding medium and sectioned on a cryostat. A TACS 2 TdT DAB (diaminobenzidine) kit (Trevigen, Gaithersburg, MD) was then used to label the sections according the manufacturer’s instructions.

## Results

### KillerRed is inducibly phototoxic and efficiently kills cells

KillerRed is a dimeric red fluorescent protein that was engineered from the *Hydrozoa* jellyfish chromoprotein anm2CP [17]. Upon irradiation with green (540-580 nm) light, KillerRed dimerizes and generates reactive oxygen species, inducing cytotoxicity. KillerRed has been harnessed to ablate cells in a number of animal models, including studies with the zebrafish tectum [18], tumor cells in mice [19], and muscle tissue in *C. elegans* [20].

**Figure 1:**
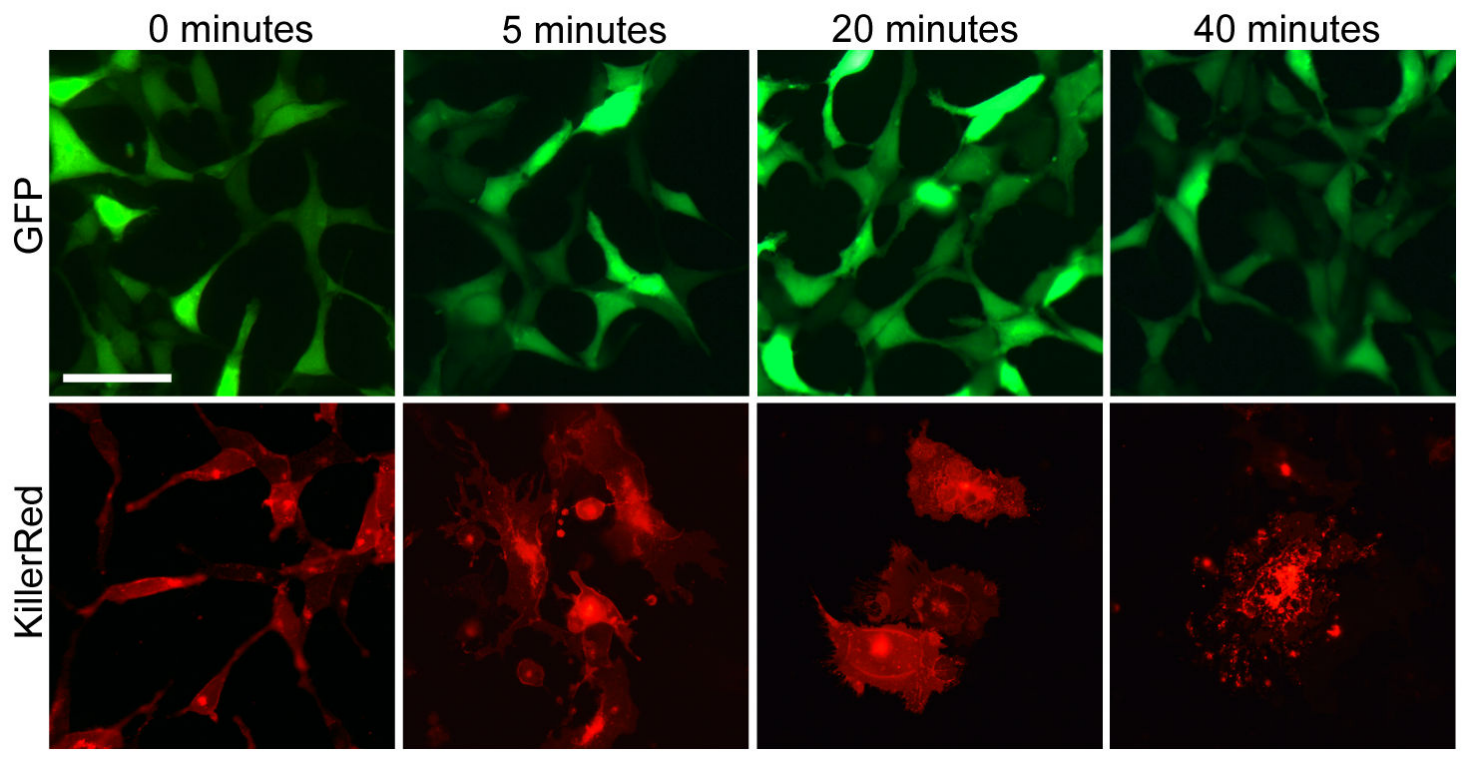
Light activated toxicity of KillerRed. An *in* *vitro* assay was performed to test the toxicity of KillerRed after exposure to a 1000 lux 540-580 nm LED light source. 293T cells transfected to express GFP showed no change after exposure to 40 minutes of green light, while cells transfected with KillerRed had abnormal morphology after 5 minutes and were necrotic by 20 minutes. After 40 minutes of light exposure, cells expressing KillerRed were eliminated. Scale bar = 50 microns.

We first tested the inducible toxicity of KillerRed in vitro by transfecting 293T cells with cDNA encoding KillerRed-mem (which has a membrane localization signal linked to the N-terminus, resulting in targeting of the protein to cellular membranes for more efficient cell death) or GFP (Figure 1). Cells were irradiated for 5, 20, or 40 minutes with a green LED light source (~1000 lux), then fixed for imaging. After 5 minutes of irradiation cells exhibited markedly abnormal morphology, and by 20 minutes they had become necrotic. After 40 minutes nearly all of the cells expressing KillerRed had disintegrated, while GFP-expressing cells by comparison were unaffected for the duration of the experiment.

### ShH10 delivers KillerRed to Müller glia

**Figure 2:**
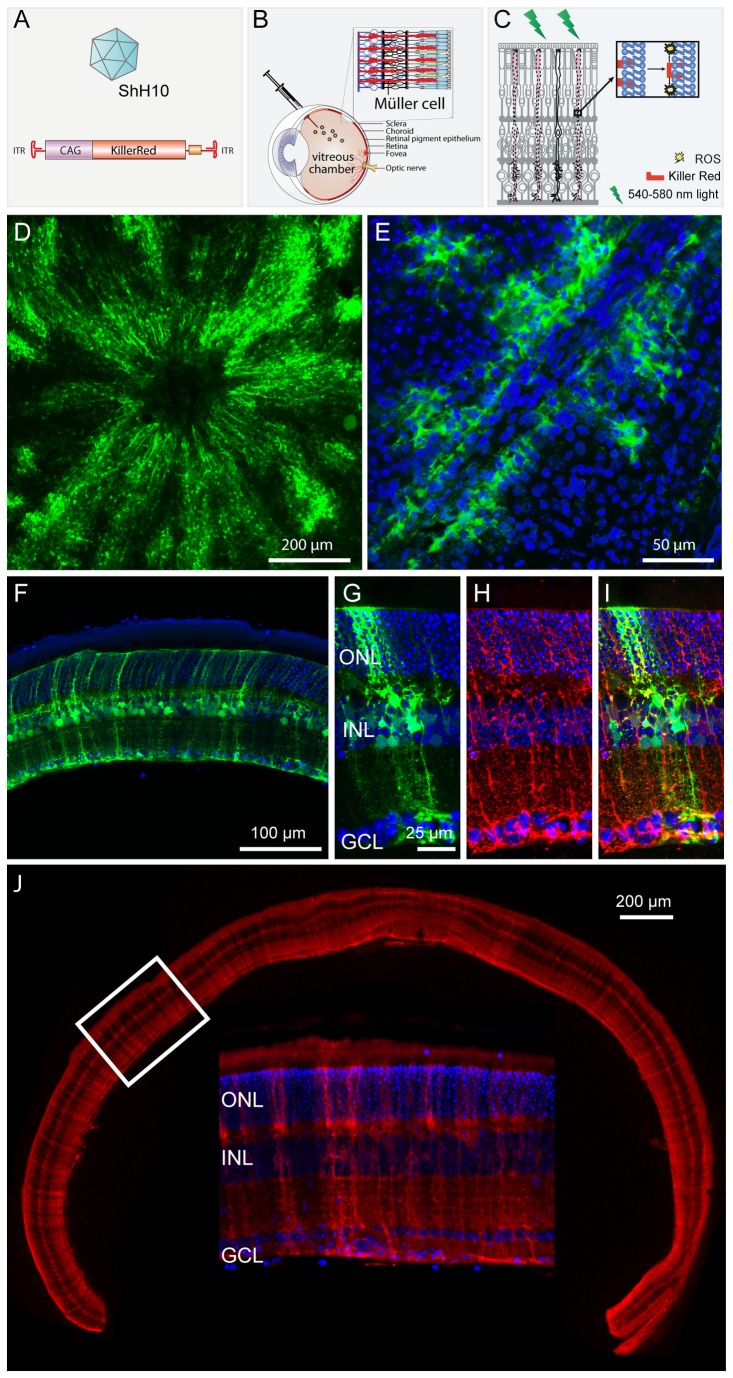
Experimental design. A) cDNA encoding KillerRed was packaged in the AAV variant ShH10, which infects Müller glia specifically. A ubiquitous CAG promoter was used to drive expression of the protein. B) ShH10-KillerRed (or ShH10-GFP in the contralateral control eye) was injected into the vitreous cavity of mouse eyes, leading to expression of KillerRed in Müller glia across the retina. C) Mice were exposed to 540-580 nm light. The KillerRed protein, which contains a membrane localization signal, dimerizes in response to green light and releases reactive oxygen species, leading to necrosis of Müller glia expressing the protein, presumably through lipid oxidation. D) A flatmount of ShH10-scCAG-GFP-injected retina, ganglion cell side up, shows that intravitreal injection of the ShH10 variant led to high levels of transgene expression across the retina. E) A high resolution image a retinal flatmount injected with ShH10-GFP shows high levels of expression in Müller cells in close proximity to blood vessels. F) A cross-section of a retina injected with ShH10-GFP shows large numbers of GFP-expressing Müller glia. G-I) ShH10-GFP and GS colocalization. ShH10-GFP injected eyes (G) labeled with anti-GS antibodies in red (H) show colocalization of GFP and GS in Müller glia in yellow (I). J) Montage of 10X images of a retina from a mouse injected with ShH10-KR and raised in the dark shows panretinal expression of KillerRed protein one month after injection. Inset depicts a higher resolution image of the boxed portion of the retina. Blue labeling is DAPI stained nuclei.

KillerRed is a small, genetically encoded protein potentially well suited for delivery via an AAV vector. Recently, we developed a novel AAV variant called ShH10 that is closely related to AAV6 and specifically and efficiently targets Müller glia when injected into the vitreous [21,22]. We hypothesized that ShH10 could be used to deliver phototoxic KillerRed to Müller glia for specific elimination of these cells. We cloned KillerRed into an AAV vector, downstream of a ubiquitous CAG promoter and flanked by inverted terminal repeats necessary for AAV packaging. This construct was then packaged into the ShH10 capsid (Figure 2A), and the ShH10-KillerRed vector was injected intravitreally into WT mice to express KillerRed in Müller glia (Figure 2B). Contralateral control eyes were injected with ShH10-GFP. One month after injection mice were exposed to ~1000 lux of 540-580 nm light, during either a single two hour session or three, one-hour sessions of light irradiation to activate KillerRed (Figure 2C). Uninjected mice were irradiated in parallel and evaluated with OCT and ERG, confirming that irradiation alone did not damage the Müller cells (data not shown).

**Figure 3:**
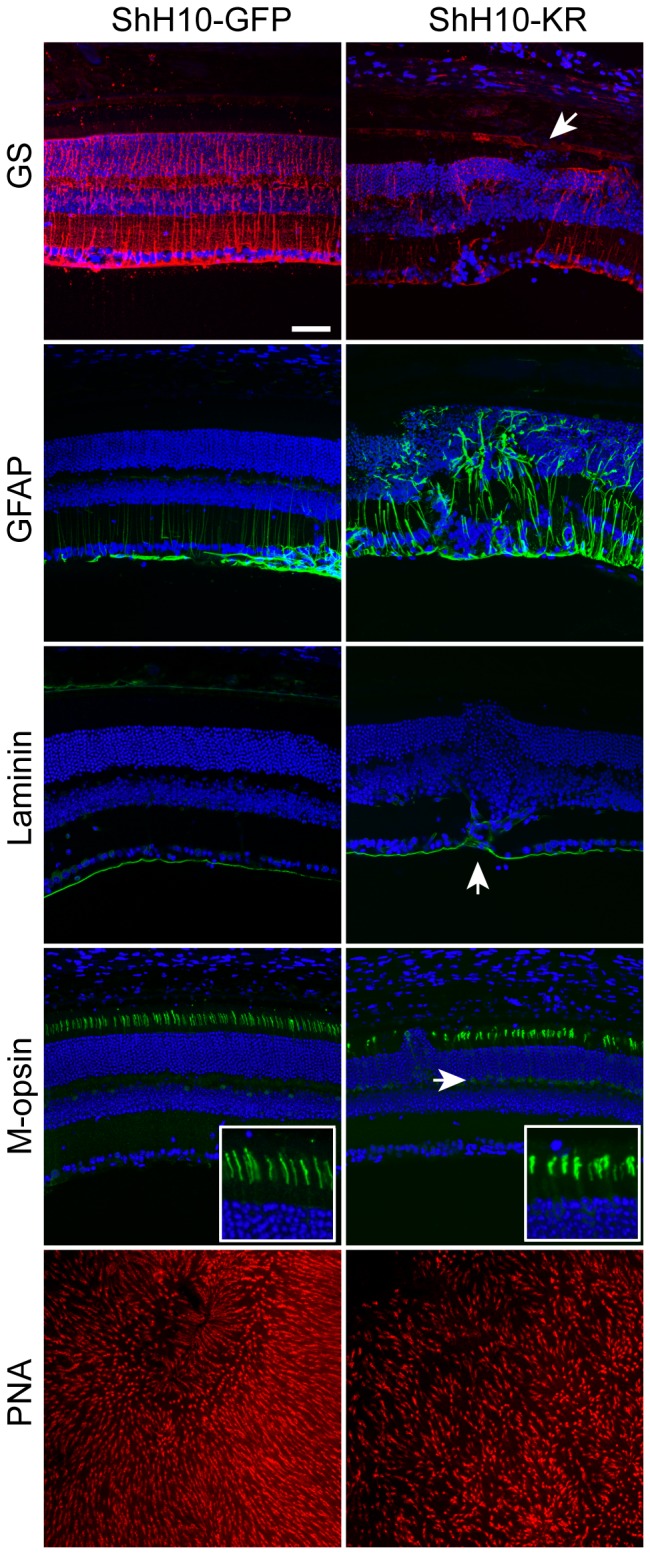
Immunohistochemical analysis of KillerRed-injected eyes. Cross-sections of agarose-embedded eyecups from animals injected with KillerRed, or contralateral control eyes injected with GFP, were harvested 3 months after illumination and labeled with antibodies against GS, GFAP, laminin, M-opsin, or PNA. DAPI, in blue, labels cell nuclei. Top row: ShH10-KillerRed-injected retinas were disorganized and had reduced anti-GS labeling. The arrow indicates an area with absence of GS labeling, and a corresponding displacement of photoreceptor cell bodies from the ONL into the subretinal space. Second row: KillerRed injected eyes had increased levels of GFAP labeling, indicating GFAP upregulation. Third row: KillerRed-injected eyes showed disorganized laminin labeling. Arrow indicates a region where laminin penetrates into the retina, near a region of disorganization of retinal layers. Fourth row: anti-cone opsin labeling shows that Killer-Red injected eyes have shortened outer segments and mislocalized opsin labeling in the axon and cone pedicle. Cone opsin labeling is absent in the area where cell bodies have penetrated into the subretinal space. Bottom row: PNA labeling shows reduced density of cones near the optic nerve head in Killer-Red injected eyes. Scale bar is 50 microns.

One month after injection, the expression of ShH10-GFP and ShH10-KillerRed in the retina were evaluated. A flatmount of a retina injected with ShH10-GFP showed that transgene expression was especially strong near the optic nerve head (Figure 2D), and that Müller cells surrounding blood vessels were strongly targeted (Figure 2E). Transverse sections of agarose-embedded sections from ShH10-GFP injected eyes revealed large numbers of Müller cells across the retina (Figure 2F) which colabeled with an antibody against GS, confirming their identity as Müller glia (Figure 2G-I). Imaging in mice injected with ShH10-KillerRed and kept in the dark to prevent induction of phototoxicity and loss of the KillerRed signal revealed that expression of KillerRed was also strong and targeted large numbers of Müller glia (Figure 3G). Amplification of the KillerRed signal and colabeling with neuronal markers confirmed that KillerRed expression was highly specific to Müller cells, with very little low-level off-target expression (Figure S1), as reported previously for ShH10 gene delivery [22]. Quantification of the loss of a Müller-specific marker following Müller cell ablation revealed a 38±8% decrease in anti-Müller cell labeling and a loss of these cells across the retina (Figure S2).

### Immunohistochemistry in KillerRed-treated eyes

ShH10-KillerRed and control ShH10-GFP-injected eyes were further analyzed by immunohistochemistry to analyze the distribution of proteins in the retina (Figure 3). Anti-GS labeling of ShH10-GFP injected eyes showed that Müller glia had normal morphology, radially spanning the retina. In contrast, there was a decrease in GS labeling of KillerRed-expressing and illuminated eyes (arrowhead), which correlated with areas of retinal disorganization and mislocalization of cells from the ONL. GFAP was markedly upregulated in ShH10-KillerRed-injected eyes, as has been shown to occur in many conditions of retinal damage or disease. GFAP labeling also revealed disorganization of Müller processes and proliferation of Müller cells. Labeling of laminin, a component of the ILM that lines the inner retina, showed occasional disruption of the retinal surface, with invasion of laminin staining into the retinal layers (arrow). Staining for M-opsin, which labels cone photoreceptors, revealed that outer segments in KillerRed-injected eyes were shortened compared to GFP-injected eyes, and labeling was absent in some areas. Labeling was also mislocalized to axons and cone pedicles (arrow). Similarly, PNA labeling of cones in retinal flatmounts, taken near the optic nerve head, showed a decrease in the density of cones.

### Histology of ShH10-KillerRed-injected eyes

**Figure 4:**
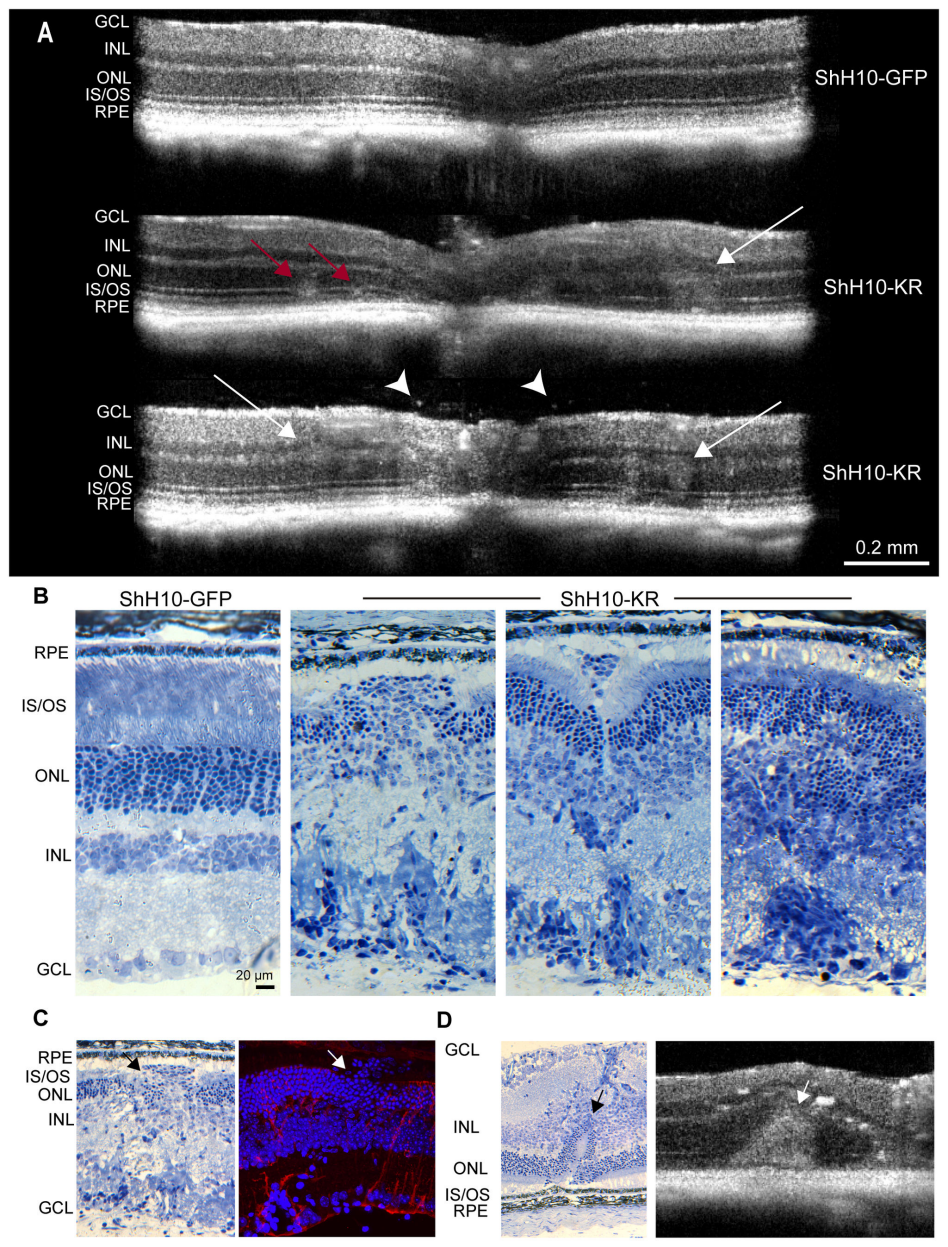
Histology of KillerRed injected eyes. A) SD-OCT imaging reveals structural abnormalities in KillerRed-injected eyes, including disorganization of retinal layers, areas of hyper-reflectivity, and retinal thinning 3 months after injection. Red arrows point to diffuse, hyperreflective regions lying between the ONL and IS/OS junction. White arrows indicate areas where retinal layers have become disorganized. White arrowheads show dense hyperreflective spots that may be macrophages in the vitreous. B) Resin-embedded thin sectioning from eyes harvested 4 months after illumination shows that KillerRed-injected eyes were characterized by a loss of retinal organization, the presence of invading cells, apparent near blood vessels, formation of rosettes, and phagocytic cells. C) Thin sections corroborate the structural changes observed by anti-GS labeling and confocal imaging. Arrows indicate absence of GS labeling coinciding with mislocalization of photoreceptor soma into the subretinal space. D) Thin sectioning shows the presence of rosette structures, which were also observed with *in* *vivo* OCT imaging.

SD-OCT *in vivo* imaging of KillerRed-injected eyes revealed structural abnormalities and disruption of retinal layers compared to GFP-expressing eyes (Figure 4A). Poorly defined hyperreflective areas, often occurring between the ONL and IS/OS, were noted in KillerRed-expressing eyes (red arrows). Blurring of the demarcations between retinal layers, including the INL, ONL, and reduced visualization of the IS/OS junctions were also observed (white arrows). Hyperreflective vitreous opacities may be inflammatory cells in the vitreous (arrowheads).

Thin (1 µm) sectioning of resin-embedded retinas confirmed the presence of structural abnormalities observed with SD-OCT (Figure 4B). Eyes injected with ShH10-GFP had normal retinal architecture, while KillerRed injected eyes were marked by disruption of retinal layers, the formation of rosette-like structures and the presence of phagocytic cells and invading inflammatory cells. Anti-IbaI and anti-Cd68 labeling confirmed the presence of macrophages in KillerRed-treated retinas (Figure S3). Structural changes were most significant in areas surrounding blood vessels and near the optic nerve head, corresponding to areas of strongest ShH10 expression, while areas in the periphery and away from regions of high expression were often more normal in appearance. These results correlate with immunohistochemistry results, including displacement of photoreceptor cell bodies outside of the ONL in areas with reduced labeling of Müller cell markers (arrows, Figure 4C) and the formation of rosettes, as observed *in vivo* (arrows, Figure 4D).

### Time course of structural changes following Müller cell ablation

**Figure 5:**
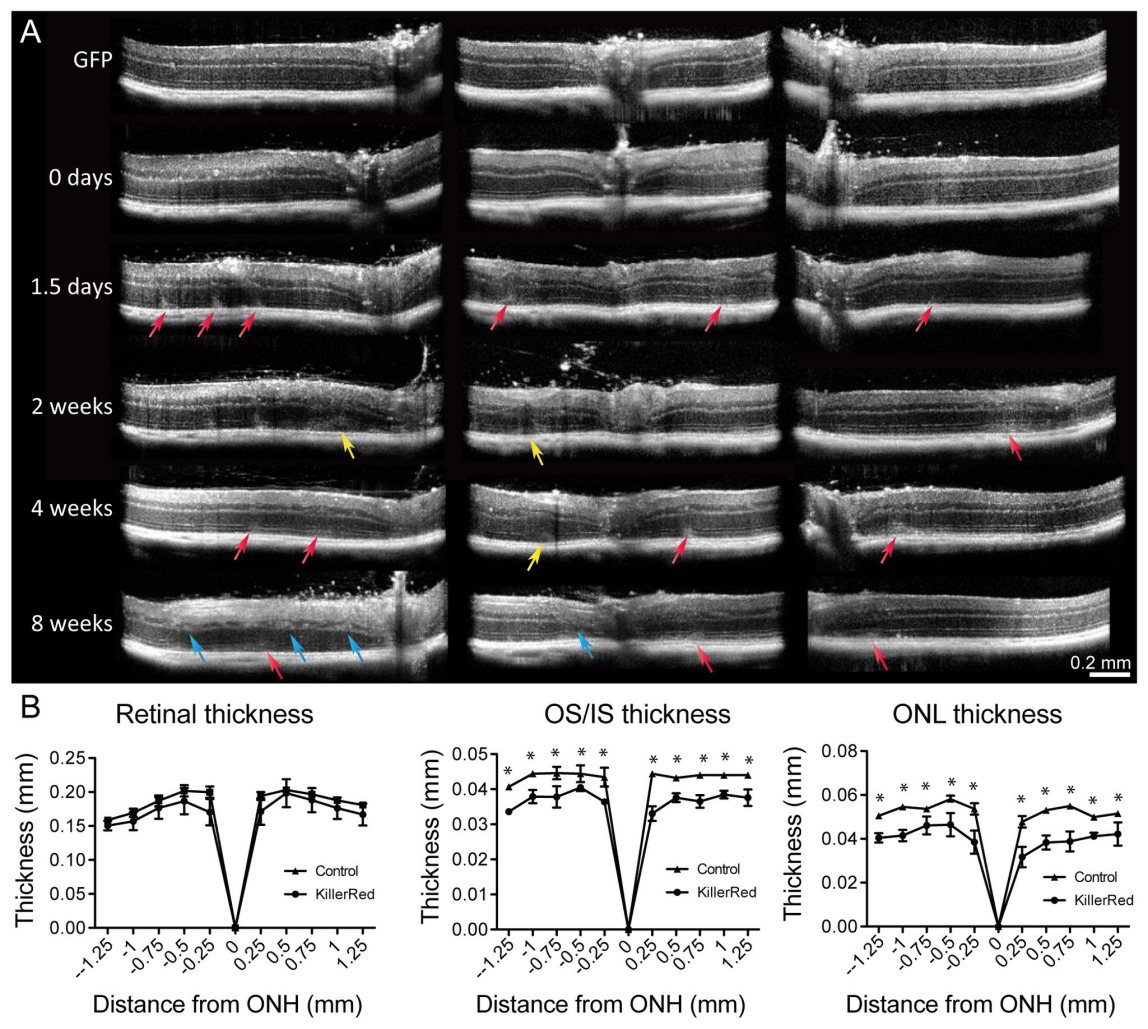
Structural changes following Müller cell ablation. A) OCT imaging collected over the course of 8 weeks reveals the progression of structural changes following a single 2 hour irradiation. Blurring of the IS/OS was visible 1.5 days after irradiation (red arrows). Disorganization of the ONL was visible 2 weeks after ablation (yellow arrows). Inner retinal changes were apparent 8 weeks after irradiation (blue arrows). B) Quantification of thickness of retinal layers from OCT images performed 5 months after irradiation (n=5) revealed that the overall thickness of the retina was only slightly (but not significantly) decreased. In contrast, a decrease in the thickness of photoreceptor inner and outer segments was significant, as was a decrease in the thickness of the ONL (*=P<0.05).

Following a single, two hour irradiation, OCT images were collected over the course of 8 weeks (n=5), revealing a progression of retinal changes that appeared to manifest first in the outer retina, followed by changes in the inner retinal layers (Figure 5A). SD-OCT images collected before irradiation showed that retinas had normal morphology, similar to GFP-injected contralateral eyes. 1.5 days after irradiation, a blurring of the IS/OS line was apparent (red arrows). Two weeks after irradiation, small retinal detachments and disorganization of the outer retinal layers were observed (yellow arrows). By 8 weeks, disorganization of the inner retina was also apparent in KillerRed-treated eyes (blue arrows).

### Retinal thickness quantification

Measurement of retinal layers in SD-OCT images showed that compared to control eyes, KillerRed retinas were only slightly thinner overall, though outer and inner segment thickness was significantly reduced (Figure 5B). Similarly, the thickness of the ONL was reduced in KillerRed-treated eyes. Photoreceptor cell death, suggested by thinning of the ONL, was confirmed using TdT DAB staining (Figure S4).

### qRT-PCR analysis of KillerRed-treated eyes

**Figure 6:**
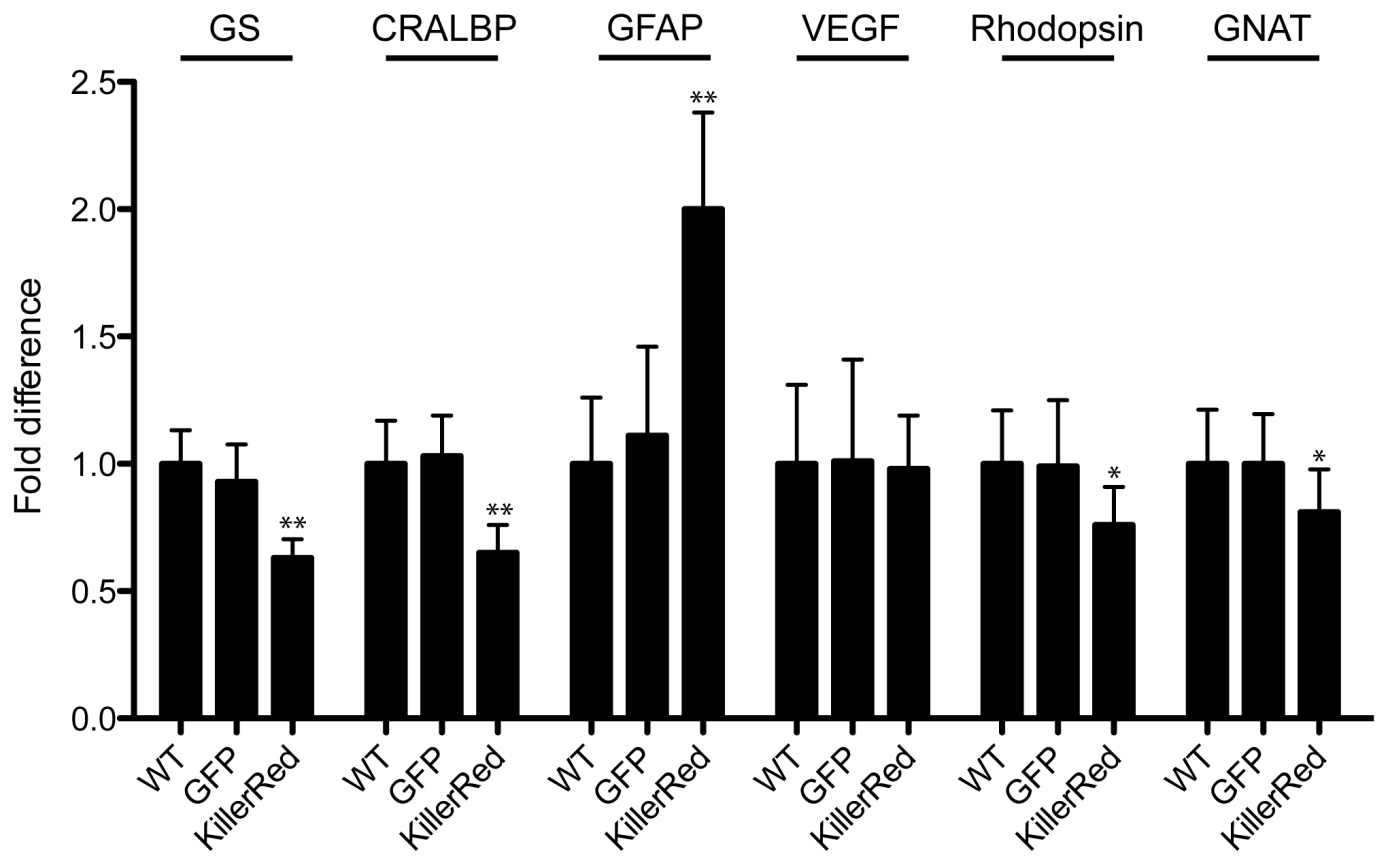
qRT-PCR analysis of mRNA levels. Quantitative RT-PCR performed on mRNA harvested from KillerRed and GFP-injected eyes, three months after irradiation, revealed that levels of GS (64% of WT), CRALBP (65%), Rhodopsin (76%), and GNAT2 (82%) were reduced in KillerRed-injected eyes compared to GFP-injected eyes or WT eyes. Levels of GFAP (205% of WT) increased, while levels of VEGF (98%) were equal to WT at the time point measured. A one-way ANOVA with a Tukey multiple comparison test was used to determine significance (*=P<0.05; ** = P<0.01).

Quantification of mRNA levels from eyes enucleated 3 months after irradiation revealed significant changes in levels of retinal transcripts in KillerRed treated eyes compared to GFP-treated, or untreated WT eyes (Figure 6). Müller cell transcripts GS (63±7% WT) and CRALBP (65±11% WT) were reduced in KillerRed treated eyes, indicative of a loss of Müller cells. In contrast, GFAP was upregulated (200±38% WT) in response to retinal injury. VEGF levels in contrast were not changed at the time point measured (98±21%). However, rhodopsin (76±15%) and GNAT (81±17%) were reduced, confirming the loss of photoreceptors previously observed histologically.

### Retinal blood vessels are altered after elimination of Müller cells

**Figure 7:**
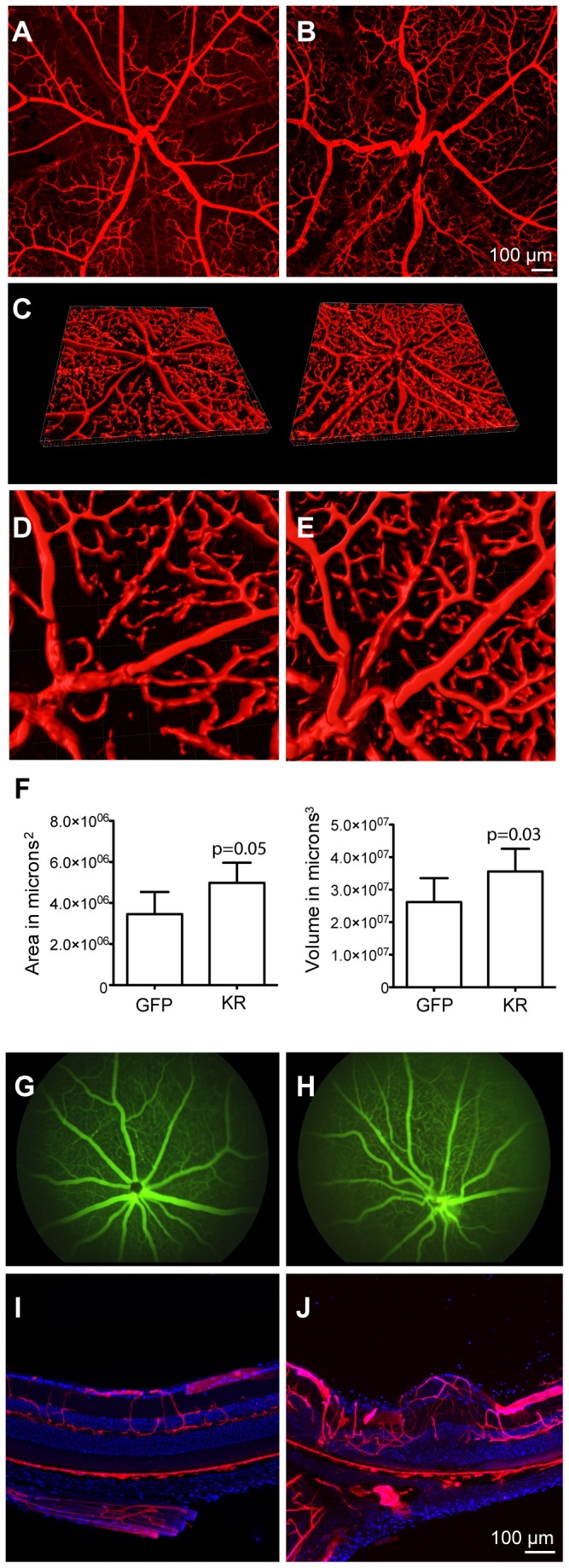
Effect of KillerRed expression on retinal vasculature. Changes in retinal blood vessels five months after irradiation were visualized in animals perfused with DiI, a lipophilic fluorescent dye that labeled endothelial cells. A) Flatmounted retina from an ShH10-GFP-injected eye, labeled with DiI. B) DiI labeled, ShH10-KillerRed injected retinal flatmount showing disorganized retinal vasculature and increasing tortuosity of blood vessels. C) 3D reconstruction, created with a confocal microscope and Imaris software, of DiI labeled retinal vasculature (left eye: GFP-injected retinal flatmount, right image: KillerRed-injected). D-E) High resolution image near the optic nerve head showing 3D reconstruction of DiI labeled blood vessels in GFP (D) or KillerRed (E) injected eyes. F) Quantification of the area and volume of retinal vasculature in DiI labeled retinal flatmounts. Killer-Red injected retinas had increased area and volume of retinal blood vessels compared to contralateral GFP-injected eyes. G-H) Fundus imaging following fluorescein injections. WT eyes have normal retinal blood vessels without leakage (G) while KillerRed-injected eyes (H) have leakage near the optic nerve head. I-J) Cross-sections through DiI labeled retinas. GFP-injected eyes show normal organization of retinal and choroidal blood vessels (I) while KillerRed-injected eyes (J) have disorganized retinal blood vessels. No significant changes in the choroidal blood supply were noted.

Müller glia line the retinal blood vessels and contribute to the formation of the blood-retinal-barrier, and the effects of Müller cell ablation on retinal vasculature were thus evaluated (Figure 7). Perfusion with the fluorescent dye DiI labels blood vessels, allowing for confocal imaging and 3D reconstruction of the vasculature. Visualization of the retinal blood vessels using this technique revealed that, compared to GFP-treated eyes (Figure 7A), KillerRed-treated eyes had significantly more tortuous retinal blood vessels and capillaries (Figure 7B). 3D reconstructions of confocal stacks further confirmed this observation (Figure 7C). In addition, high-resolution images of 3D images, taken near the optic nerve head, show in detail the formation of additional blood vessels in KillerRed-ablated eyes (Figure 7D, E). Quantification of blood vessels in 3D reconstructions showed a significant increase in the area and volume of the vasculature compared to GFP-expressing, contralateral control eyes (Figure 7F).

Fundus imaging of animals injected intraperitoneally with fluorescein dye was used to check for leakage of retinal blood vessels. Imaging in untreated WT animals shows normal conformation of retinal blood vessels and an absence of fluorescein leakage (Figure 7G). In comparison, in animals treated with KillerRed, leakage was often noted near the optic nerve head, and an increase in vascular branching and tortuosity was observed (Figure 7H).

Finally, retinas perfused with DiI were embedded in agarose and transverse sections were made. In agreement with histology, DiI and fluorescein imaging, an increase in the density of retinal blood vessels was observed, with abnormal morphology of blood vessels accompanying disorganization of retinal layers (Figure 7I,J).

### Retinal function is altered after ablation of Müller glia

**Figure 8:**
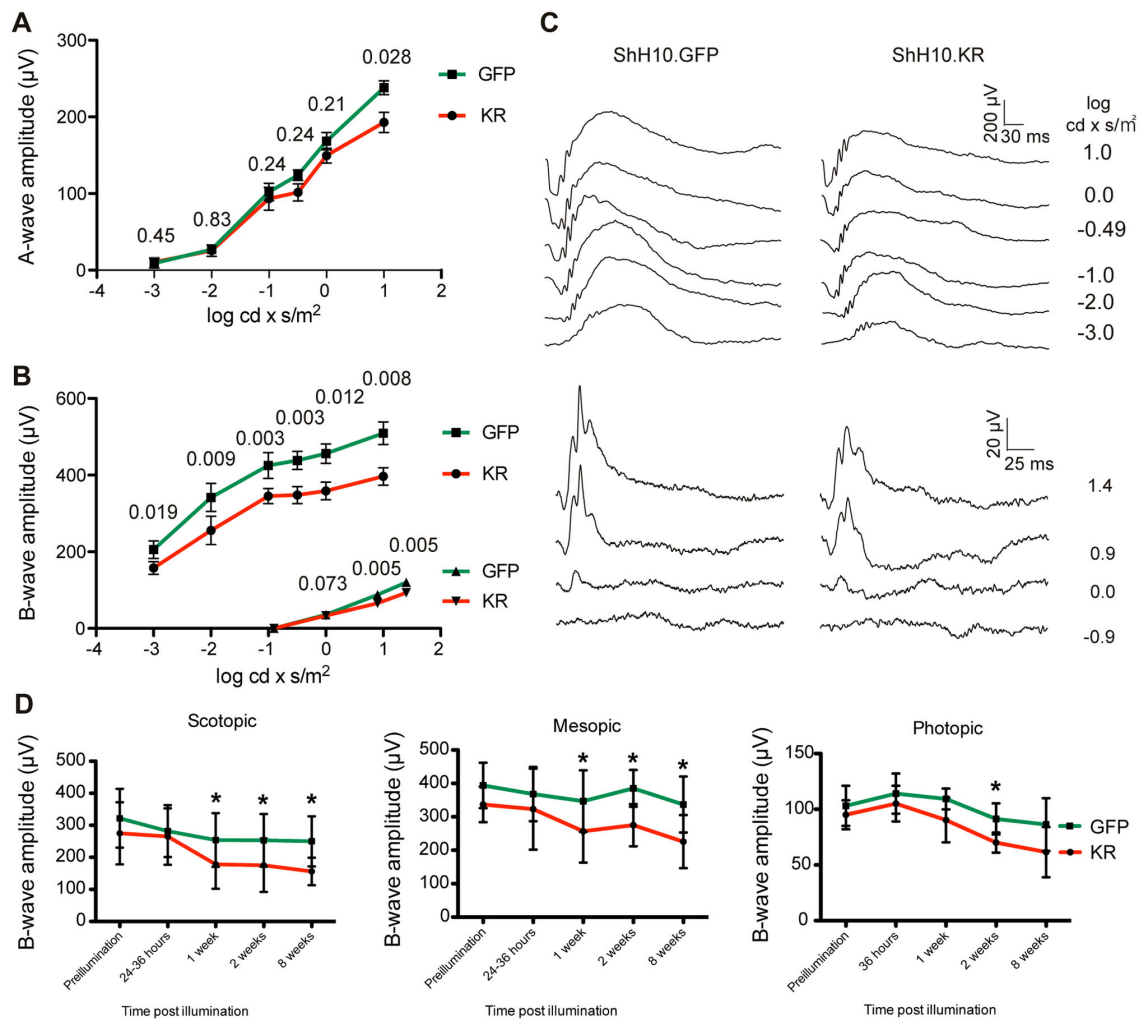
Electroretinogram recordings. A) Graph of the amplitude of scotopic, full field a-waves in response to flashes of increasing intensity, recorded 4 months after irradiation. A-wave amplitudes were reduced in KillerRed-injected eyes. The reduction in a-wave amplitude was statistically significant, though only at the highest light intensity tested. P-values from a 2-tailed paired Student’s t-test are indicated above the graph for each light intensity. B) Graph of the amplitude of scotopic (upper traces) and photopic (lower traces) full field b-waves in response to flashes of increasing intensity. B-wave amplitudes were significantly decreased in KillerRed injected eyes for every light intensity under both photopic and scotopic conditions. C) Representative ERG traces from ShH10-GFP and ShH10-KillerRed injected eyes under scotopic (upper sets of traces) and photopic (lower sets) conditions. D) ERG recordings made under rod-specific conditions (0.01 log cd x s/m^2^), rod and cone mixed signal conditions (1 log cd x s/m^2^), or light adapted cone-mediated conditions (10 log cd x s/m^2^) were recorded over the course of 8 weeks. This time course of functional loss revealed that the rod-driven signal was significantly reduced 1 week after irradiation. Similarly, under mesopic conditions, a decrease was observed one week after irradiation. The photopic, light-adapted ERG was decreased 2 weeks after Müller cell ablation.

ERGs record the change in the electrical potential of the retina in response to a flash of light, and a change in the amplitude of the ERG waveform is indicative of functional changes in the retina. Recordings from KillerRed injected eyes (n=10) revealed a reduction in the scotopic (or dark-adapted) and photopic (light adapted, cone-mediated) ERG (Figure 8). Across a range of light intensities, the amplitude of the scotopic ERG a-wave, which arises from photoreceptors, was reduced in KillerRed-expressing eyes compared to contralateral control eyes injected with ShH10-GFP, though the change was only significant at the highest light intensity measured (Figure 8A). In contrast, the scotopic ERG b-wave was significantly reduced at all light intensities (Figure 8B). A reduction of the b-wave indicates functional changes that occur in the inner retina [23]. Significant reductions were also observed in photopic ERG amplitudes (lower traces, Figure 8B). Representative ERG waveforms (Figure 8C) illustrate the changes observed across the range of light intensities tested under scotopic (top traces) or photopic (bottom traces) conditions.

A time course of ERG recordings was performed to determine the rate of functional loss in the retina following Müller cell ablation (Figure 8D). Scotopic rod-driven ERGs, mesopic ERGs arising from rod and cone signals and light adapted ERGs arising from cones were recorded from animals injected with KillerRed and GFP in the contralateral eye following a single 2 hour irradiation over the course of 8 weeks. A significant decrease in the rod-driven and rod+cone-driven ERG was recorded one week after irradiation, while a significant difference in the cone-mediated ERG was recorded 2 weeks after irradiation.

## Discussion

Administration of ShH10-KillerRed selectively eliminates Müller glia in the mouse retina, resulting in changes to retinal neurons and vasculature similar to histopathological features seen in donor retinas of MacTel-2 patients. Müller cell loss was associated with dramatic alterations in retinal structure, including damage to photoreceptors, characterized by mislocalization of opsin, reduction in the density of cones, a decrease in photoreceptor-specific transcripts and a reduction in the amplitude of the scotopic and photopic ERG. Photoreceptors cell death was confirmed by labeling for markers of apoptosis. These results mimic those reported recently demonstrating loss of photoreceptors in MacTel-2 patients [24]. Interestingly, a blurring of the IS/OS line was visible in OCT images collected 1.5 days after Müller cell ablation. Loss of the OLM, which is formed by adherens junctions with outer Müller cell endfeet, coincided with displacement of photoreceptors into the subretinal space. These changes are consistent with the important role Müller cells play in supporting photoreceptors. Vascular changes, including increased permeability of the blood-retina-barrier, and proliferation of blood vessels, also followed Müller cell ablation.

Recently, evidence suggesting that the idiopathic disease MacTel-2 is associated with dysfunction in Müller cells has emerged. Symptoms of MacTel-2 include the formation of telangiectatic blood vessels and vascular leakage, foveal atrophy and reduced retinal transparency. Although it is unclear which cell types are responsible for the underlying deficiency in MacTel-2, several recent studies have offered evidence for Müller cell involvement. For example, a recent histological study of an eye from a MacTel-2 patient showed loss of Müller cell markers including vimentin and GS in the macula, suggesting that Müller cell loss plays a role in the etiology of this disease [5]. In addition, many of the characteristics of MacTel-2 can be recapitulated in mice by the genetic ablation of Müller cells [11].

The loss of macular pigment in the fovea is also observed in MacTel-2 patients. The fovea, a region of the primate retina used for high acuity vision, contains cone photoreceptors, as well as a zone of specialized Müller glia called the Müller cell cone. Processes of Müller cells in this region may store macular pigment, as suggested by a recent study in which primate photoreceptors were ablated but macular pigment remained unchanged [10]. In contrast, areas of the human MacTel-2 eye lacking Müller cells corresponded to areas of macular pigment loss [25].

The changes observed after Müller cell loss in this animal model mimic some of the alterations observed in MacTel-2 patients and in previous models of Müller cell depletion [9,11]. In particular, we observed a reduction in GS expression in regions of KillerRed expression corresponding to retinal atrophy, disorganization of the retina, changes in inner and outer photoreceptor segments in OCT imaging [26] and reduction in the ERG [27]. However, subretinal neovascularization, which is also seen in MacTel-2 patients, was not observed at the time points tested. It may be interesting to explore whether delivery of a higher number of viral particles leading to greater numbers of eliminated Müller cells results in additional retinal changes, such as subretinal neovascularization.

One important aspect of this study is that this approach may be used to create a large animal model of Müller cell loss. Although several methods have been used to create rodent models of Müller cell disease, murine models have several drawbacks including a lack of macular pigment, and no large animal model exists. There are important physiological differences between the eyes of mice and humans, including the presence of a macula and Müller cell cone. AAV, injected intravitreally in the non-human primate eye, can infect Müller cells in the fovea [28], and therefore in future studies injections of ShH10 may be used to eliminate foveal Müller cells in the primate, which may represent an important step forward in the understanding of Müller cell involvement in MacTel-2 and other retinal degenerative diseases. It may be possible to modify this approach by increasing or decreasing the number of viral particles that are delivered, as well as the timing and duration of irradiation in order to control the number of Müller cells that are eliminated. In addition, once the mechanism of MacTel-2 is better understood, ShH10 could be used to mediate shRNA or targeted DNA nuclease expression to knock down genes involved in the disease, or to overexpress a therapeutic molecule.

## Supporting Information

Figure S1 **Müller cell specificity of ShH10.** Colabeling with neuronal markers and amplification of KillerRed or GFP revealed that ShH10 was highly specific to Müller cells with little off-target expression. A-B) Colabeling of retinas injected with ShH10-KillerRed. C-N) Colabeling of retinas injected with ShH10-GFP. Insets are high-resolution images of labeling. A) In retinal flatmounts mounted photoreceptor side up, labeling for KillerRed and S-opsin showed the absence of KillerRed expression in photoreceptor outer segments. B) In retinal flatmounts mounted ganglion side up anti-KillerRed labeling showed the presence of very few KillerRed positive ganglion cells. C-E) Confocal stack imaging of cross-sections from ShH10-GFP-injected eyes, colabeled with anti-GFP (C) and anti-calbindin (D) antibodies. (E) Image shows overlay of signal from the colabeling. F-H) Anti-GFP and anti-PKCα labeling. I-K) Anti-GFP and anti-NeuN colabeling. Very few GFP-positive ganglion cells were observed (approximately 4% of infected cells, as previously reported). Arrowheads indicate the presence of one such colabeled ganglion cell. L-N) Anti-GFP and anti-M-opsin labeling. Scale bar in A-B = 50 µM. Scale bar in C-N = 50 µM.
(TIF)

Figure S2 **Loss of Müller cells following ablation.** Agarose embedded and sectioned eyes labeled for the Müller cell-specific protein glutamine synthetase revealed a loss of Müller cells 8 weeks after KillerRed-mediated ablation. While untreated WT eyes and GFP-injected eyes had normal and regular staining of Müller cells, KillerRed retinas showed loss of Müller cell markers across the retina, corresponding to loss of structural integrity. Quantification of the loss of Müller cell labeling revealed a significant decrease in labeling (38±8%, P=0.007) compared to WT or GFP-treated eyes. Higher resolution images show detail of anti-GS labeling.
(TIF)

Figure S3 **Presence of inflammatory cells in KillerRed-treated eyes.** Labeling of markers for macrophages and microglia showed the presence of invading inflammatory cells in ShH10-KillerRed-injected retinas, but not in GFP-treated contralateral eyes. Blue staining: DAPI; red labeling: anti-IbaI; yellow labeling, anti-Cd68.
(TIF)

Figure S4 **Cell death in the ONL following Müller cell ablation.** Labeling of apoptotic cells using a TACS 2 TdT diaminobenzidine kit revealed the presence of dying cells in the ONL, corresponding to the thinning of the ONL observed using OCT.
(TIF)
