# Supplementary figures and images for: AAV-Mediated, Optogenetic Ablation of Müller Glia Leads to Structural and Functional Changes in the Mouse Retina
Source: PLoS One. 2013 Sep 27;8(9):e76075. doi: 10.1371/journal.pone.0076075 (PMC3785414; doi:10.1371/journal.pone.0076075)

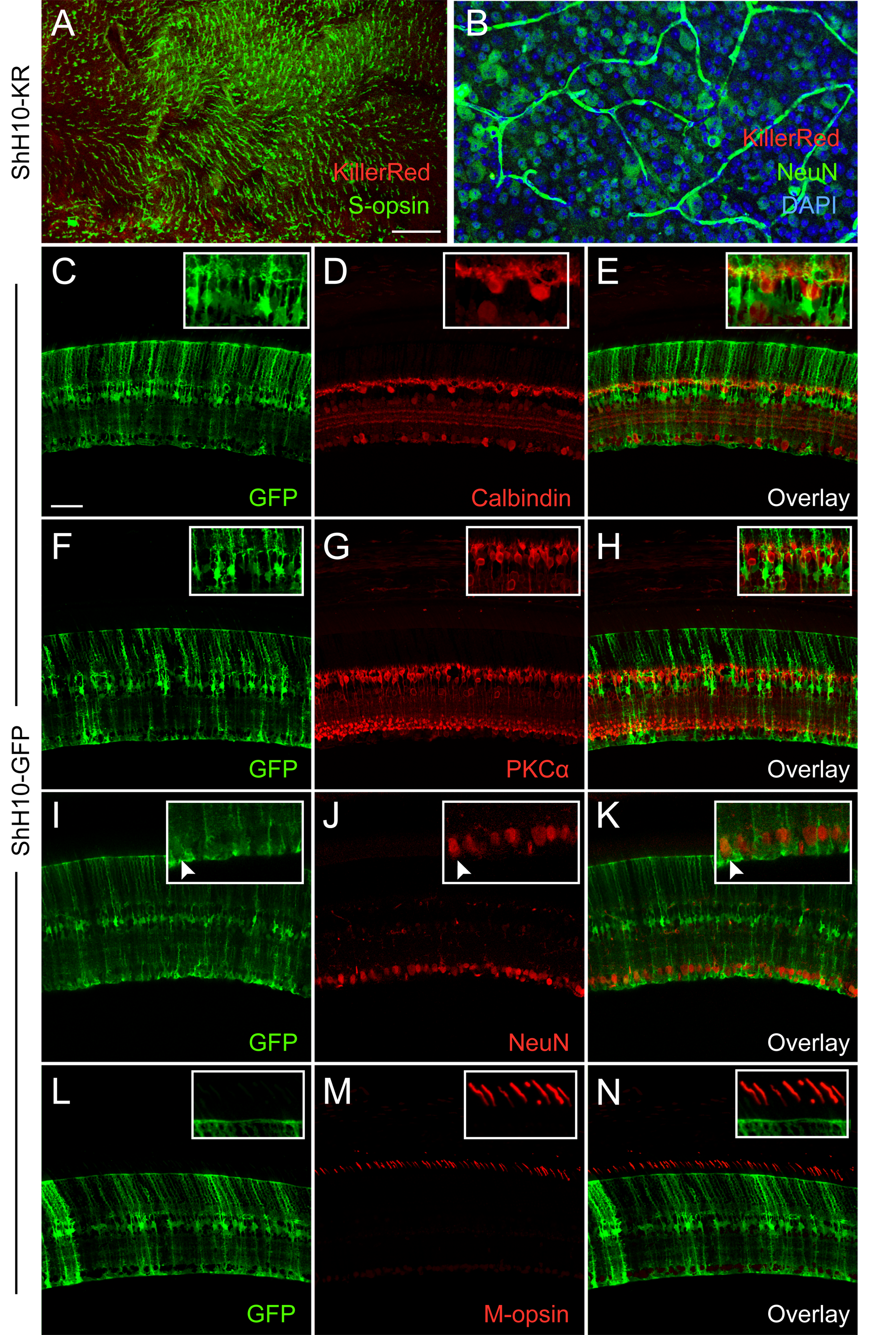

Supplement: Figure S1 — Müller cell specificity of ShH10. Colabeling with neuronal markers and amplification of KillerRed or GFP revealed that ShH10 was highly specific to Müller cells with little off-target expression. A-B) Colabeling of retinas injected with ShH10-KillerRed. C-N) Colabeling of retinas injected with ShH10-GFP. Insets are high-resolution images of labeling. A) In retinal flatmounts mounted photoreceptor side up, labeling for KillerRed and S-opsin showed the absence of KillerRed expression in photoreceptor outer segments. B) In retinal flatmounts mounted ganglion side up anti-KillerRed labeling showed the presence of very few KillerRed positive ganglion cells. C-E) Confocal stack imaging of cross-sections from ShH10-GFP-injected eyes, colabeled with anti-GFP (C) and anti-calbindin (D) antibodies. (E) Image shows overlay of signal from the colabeling. F-H) Anti-GFP and anti-PKCα labeling. I-K) Anti-GFP and anti-NeuN colabeling. Very few GFP-positive ganglion cells were observed (approximately 4% of infected cells, as previously reported). Arrowheads indicate the presence of one such colabeled ganglion cell. L-N) Anti-GFP and anti-M-opsin labeling. Scale bar in A-B = 50 µM. Scale bar in C-N = 50 µM. (TIF) [file pone.0076075.s001.tif]

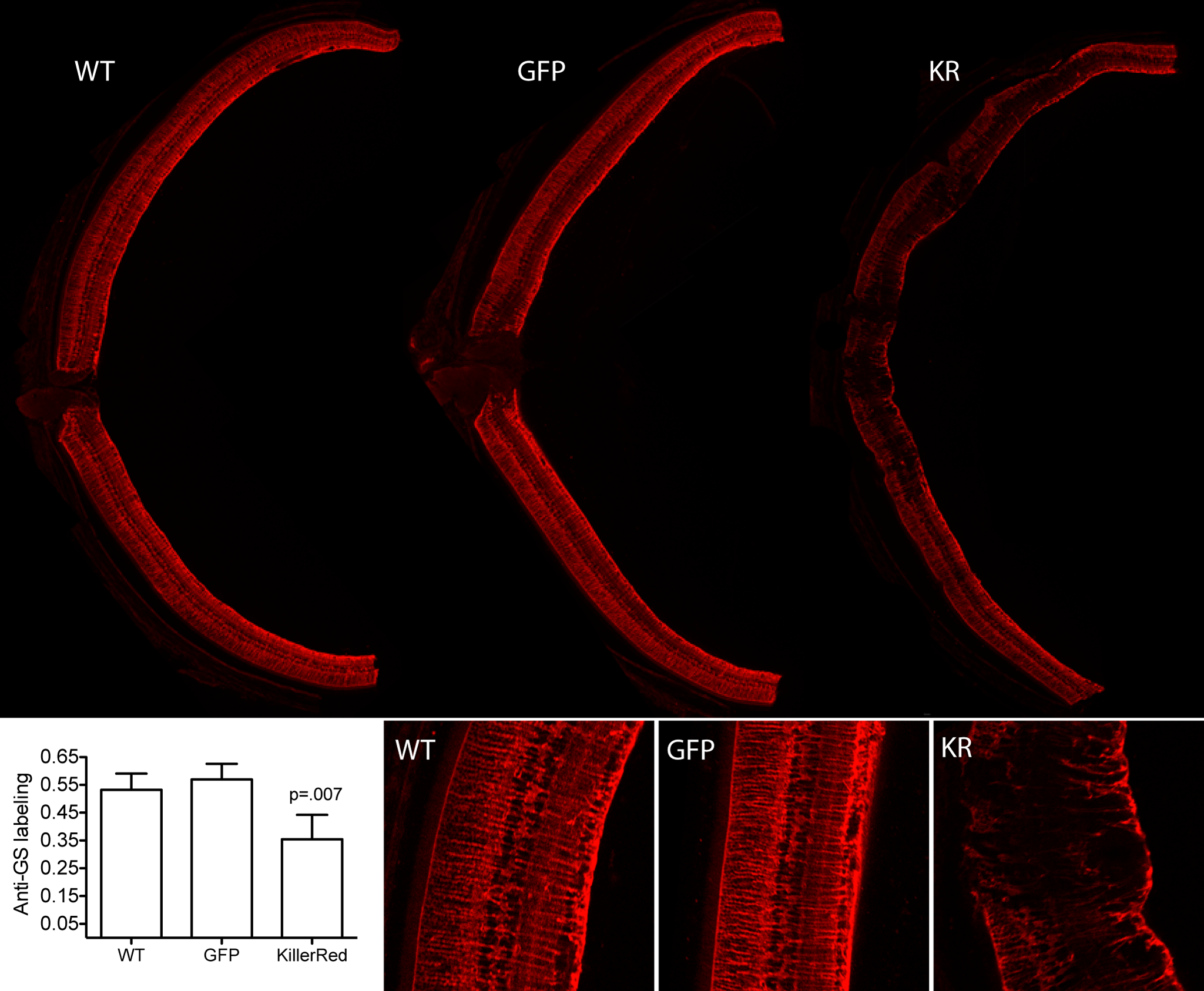

Supplement: Figure S2 — Loss of Müller cells following ablation. Agarose embedded and sectioned eyes labeled for the Müller cell-specific protein glutamine synthetase revealed a loss of Müller cells 8 weeks after KillerRed-mediated ablation. While untreated WT eyes and GFP-injected eyes had normal and regular staining of Müller cells, KillerRed retinas showed loss of Müller cell markers across the retina, corresponding to loss of structural integrity. Quantification of the loss of Müller cell labeling revealed a significant decrease in labeling (38±8%, P=0.007) compared to WT or GFP-treated eyes. Higher resolution images show detail of anti-GS labeling. (TIF) [file pone.0076075.s002.tif]

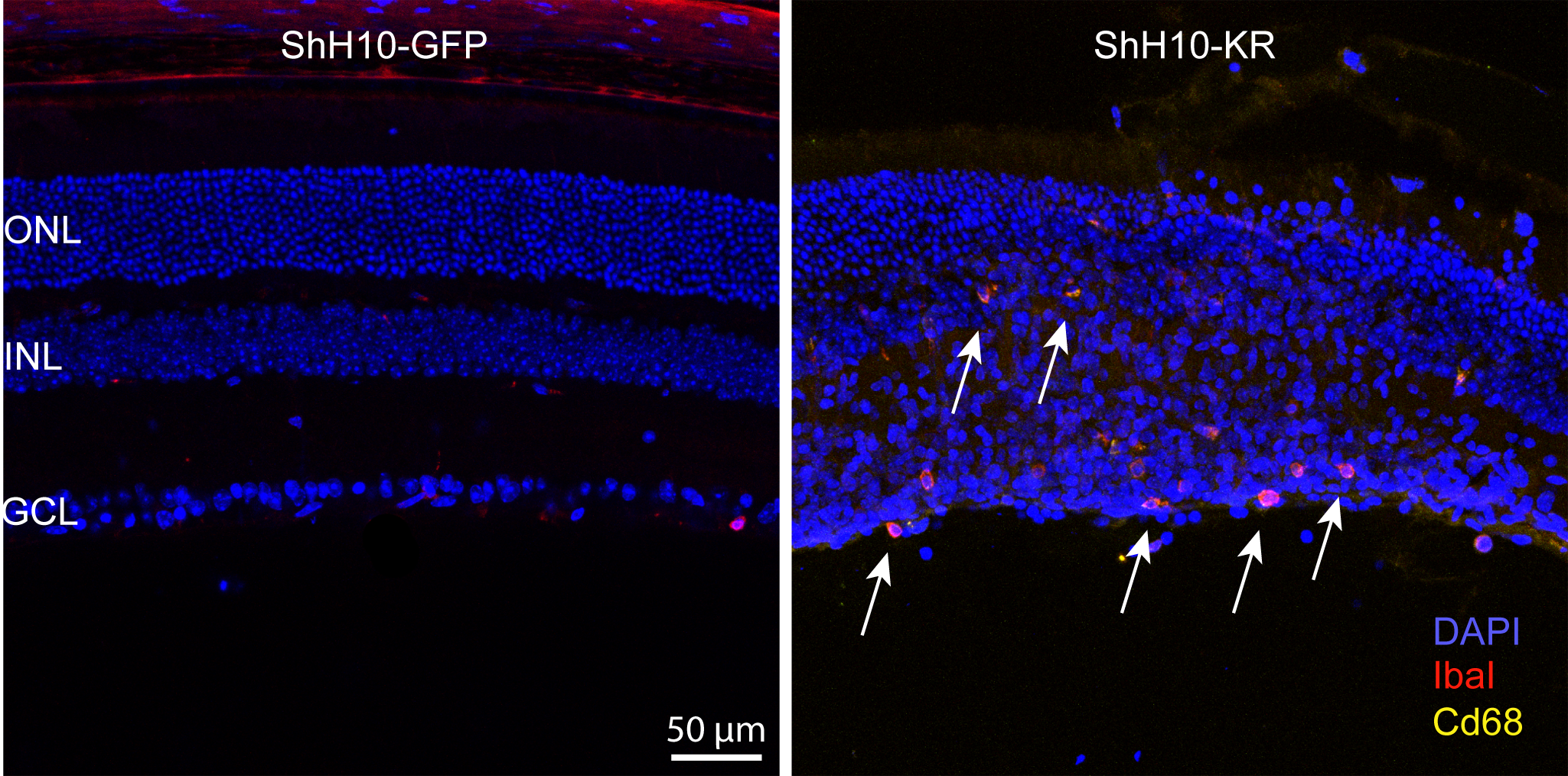

Supplement: Figure S3 — Presence of inflammatory cells in KillerRed-treated eyes. Labeling of markers for macrophages and microglia showed the presence of invading inflammatory cells in ShH10-KillerRed-injected retinas, but not in GFP-treated contralateral eyes. Blue staining: DAPI; red labeling: anti-IbaI; yellow labeling, anti-Cd68. (TIF) [file pone.0076075.s003.tif]

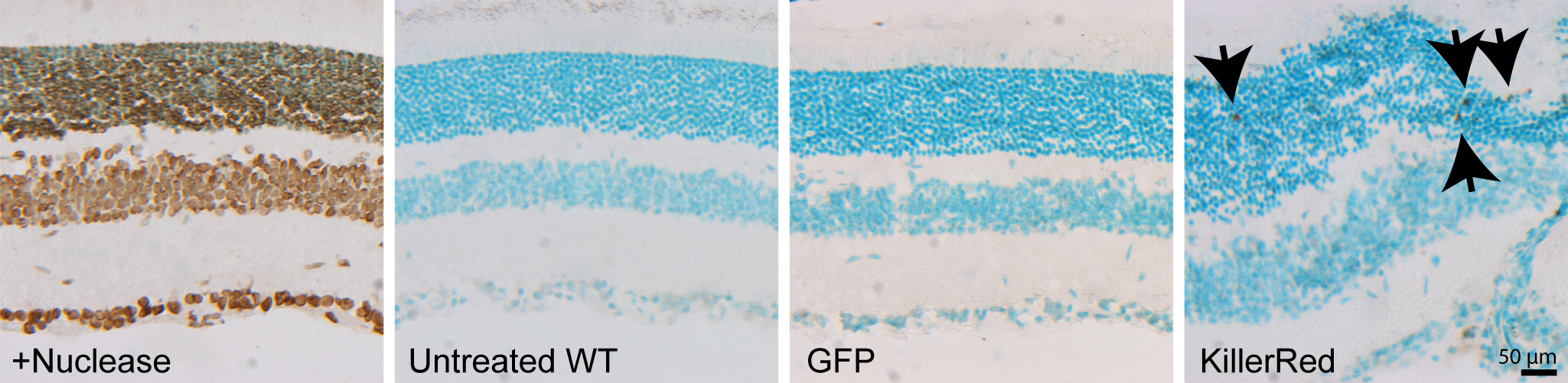

Supplement: Figure S4 — Cell death in the ONL following Müller cell ablation. Labeling of apoptotic cells using a TACS 2 TdT diaminobenzidine kit revealed the presence of dying cells in the ONL, corresponding to the thinning of the ONL observed using OCT. (TIF) [file pone.0076075.s004.tif]
